# The Petri Dish-N2B27 Culture Condition Maintains RPE Phenotype by Inhibiting Cell Proliferation and mTOR Activation

**DOI:** 10.1155/2020/4892978

**Published:** 2020-08-13

**Authors:** Hui Lou, Chunpin Lian, Fanjun Shi, Liqun Chen, Sicheng Qian, Hui Wang, Xiaoyun Zhao, Xiaoyan Ji, Jingfa Zhang, Guoxu Xu

**Affiliations:** ^1^Department of Ophthalmology, The Second Affiliated Hospital of Soochow University, Suzhou 215004, China; ^2^Department of Regenerative Medicine and Stem Cell Research Center, Tongji University School of Medicine, Shanghai 200092, China; ^3^Department of Ophthalmology, The Second People's Hospital of Hefei, Hefei 230011, China; ^4^Department of Ophthalmology, Shanghai General Hospital (Shanghai First People's Hospital), Shanghai Jiao Tong University, Shanghai 200080, China; ^5^National Clinical Research Center for Eye Diseases, Shanghai Key Laboratory of Ocular Fundus Diseases, Shanghai Engineering Center for Visual Science and Photomedicine, Shanghai Engineering Center for Precise Diagnosis and Treatment of Eye Diseases, Shanghai 200080, China

## Abstract

**Objective:**

To develop a method for the rapid isolation of rat RPE cells with high yield and maintain its epithelial state in modified culture system.

**Methods:**

The eyeballs were incubated with dispase. The retina was isolated with RPE attached and cut into several pieces. Following a brief incubation in growth medium, large RPE sheets can be harvested rapidly. RPE cells were divided into four groups and cultured for several weeks, that is, (1) in cell culture dishes with 10% FBS containing medium (CC dish-FBS), (2) in petri dishes with 10% FBS containing medium (Petri dish-FBS), (3) in cell culture dishes with N2 and B27 containing medium (CC dish-N2B27), and (4) in petri dishes with N2 and B27 containing medium (Petri dish-N2B27). Morphological and biological characteristics were investigated using light microscopy, Q-PCR, and western blot.

**Results:**

The retina would curl inwardly during the growth medium incubation period, releasing RPE sheets in the medium. Compared with low density group (5,000 cells/cm^2^), RPE cells plated at high density (15,000 cells/cm^2^) can maintain RPE morphology for a more extended period. Meanwhile, plating RPE cells at low density significantly reduced the expression of RPE cell type-specific genes (RPE65, CRALBP, and bestrophin) and increased the expression of EMT-related genes (N-cadherin, fibronectin, and *α*-SMA), in comparison with the samples from the high density group. The petri dish culture condition reduced cell adhesion and thus inhibited RPE cell proliferation. As compared with other culture conditions, RPE cells in the petri dish-N2B27 condition could maintain RPE phenotype with increased expression of RPE-specific genes and decreased expression of EMT-related genes. The AKT/mTOR pathway was also decreased in petri dish-N2B27 condition.

**Conclusion:**

The current study provided an alternative method for easy isolation of RPE cells with high yield and maintenance of its epithelial morphology in the petri dish-N2B27 condition.

## 1. Introduction

The retinal pigment epithelium (RPE), located between the neural retina and the choroid, is a polarized monolayer of cobblestone-like cells. The RPE plays many critical functions in the eye, including secretion of nutrients and growth factors, phagocytosis of photoreceptor outer segments, formation of the blood-retinal barrier, and modulating the immune response of the eye [1, 2]. Alterations of RPE cell morphology and function are common features shared by many retinal diseases (RD) such as retinitis pigmentosa and age-related macular degeneration [3]. Located at the back of the eye, the RPE is difficult to access *in vivo*, making the direct observation and experimentation of RPE problematic. Therefore, it is necessary to establish natural-type RPE cell cultures to study RPE cell biology and function in a flexible and controlled environment [4].

Decades of research on the biological functions of RPE cells have led to novel developments in our understanding of RD pathogenesis. Besides primary RPE cultures, a variety of established RPE cell lines such as ARPE-19 and RPE-J are also used [5]. However, transformed or immortalized RPE cell lines differ in many respects from natural-type RPE cells. For example, ARPE-19 cells are often characterized by losing of RPE cell type-specific features and acquiring mesenchymal cell-like properties. Besides, they also possessed low transepithelial electrical resistance and decreased expression of RPE-selective genes [6, 7], limiting its potential use to unveil the pathogenesis of RD. Comparing the immortalized cell lines, natural-type RPE cell cultures obtained from eye tissues provide a valuable model for the study of diseases involving RPE pathology.

Cultured RPE cells can be used to study RPE cell type-specific functions and basic RPE cell biology, including epithelial cell polarity, lysosomal homeostasis, and autophagy [2, 8–11]. Over the decades, a wide range of methods for RPE cell isolation and culture have been reported, suggesting a growing interest in this area of research [12, 13]. A number of protocols regarding the isolation and culture of RPE cells from rats were published [14]. However, very few methods described the detailed procedure of the isolation and culture of RPE cells from albino rats. And the isolation of RPE cells from adult rats still remains a challenge nowadays due to the interdigitation of RPE apical microvilli with the photoreceptor outer segments and the attachment of RPE cells to Bruch's membrane. In addition, the variation of enzyme concentration and incubation time also results in inconsistent cell yield and cell viability. Furthermore, *in vitro* cultured RPE cells gradually lose epithelial characteristics and spontaneously undergo epithelial-mesenchymal transition (EMT), especially in serum-based culture conditions [15]. Serum-based medium contains many unknown factors and hormones, which may affect cell morphology and cell development. Therefore, many researchers tried to find a defined and serum-free culture system that could inhibit EMT.

To provide an easy method for the rapid isolation of rat RPE cells with high yield, we modified the isolation method of primary RPE cells with the combination of enzymatic digestion and mechanical dissection. And to maintain its epithelial state and inhibit EMT, we optimize the culture system with DMEM/F12 supplemented with N2 and B27 in the petri dish. N2 and B27 supplements are serum-free and contain many factors of great importance for the maintenance of RPE phenotype [16, 17], while the petri dishes reduce cell adhesion and spreading and thus inhibit cell proliferation. The combination of these two conditions is sufficient to maintain RPE phenotype. The present study showed that this could be an alternative method with easy manipulation for RPE isolation and *in vitro* culture, facilitating its further study for the pathogenesis of RD.

## 2. Materials and Methods

### 2.1. Reagents and Antibodies

Dispase was purchased from Roche (Shanghai, China). N2 and B27 supplements were purchased from Gibco (Shanghai, China). Primary antibodies against phospho-mTOR (2971), total-mTOR (4517S), phospho-p70S6K (9208), total-p70S6K (9202), phospho-AKT (9272), and total-AKT (9271) were from Cell Signaling Technology (Danvers, MA, USA); the antibody against GAPDH (G9545) was purchased from Sigma-Aldrich (St. Louis, MO, USA).

### 2.2. Animals

Dark Agouti (DA) rats (10 days old) and Sprague-Dawley (SD) rats (10 days old) were purchased from Shanghai Laboratory Animal Co. (Shanghai, China). They were bred on a 12:12-hour light and dark cycle, with the light cycle occurring during daytime. The rats were treated in accordance with the ARVO Statement for the Use of Animals in Ophthalmic and Vision Research.

### 2.3. Isolation and Culture of Rat RPE Cells

**Figure 1:**
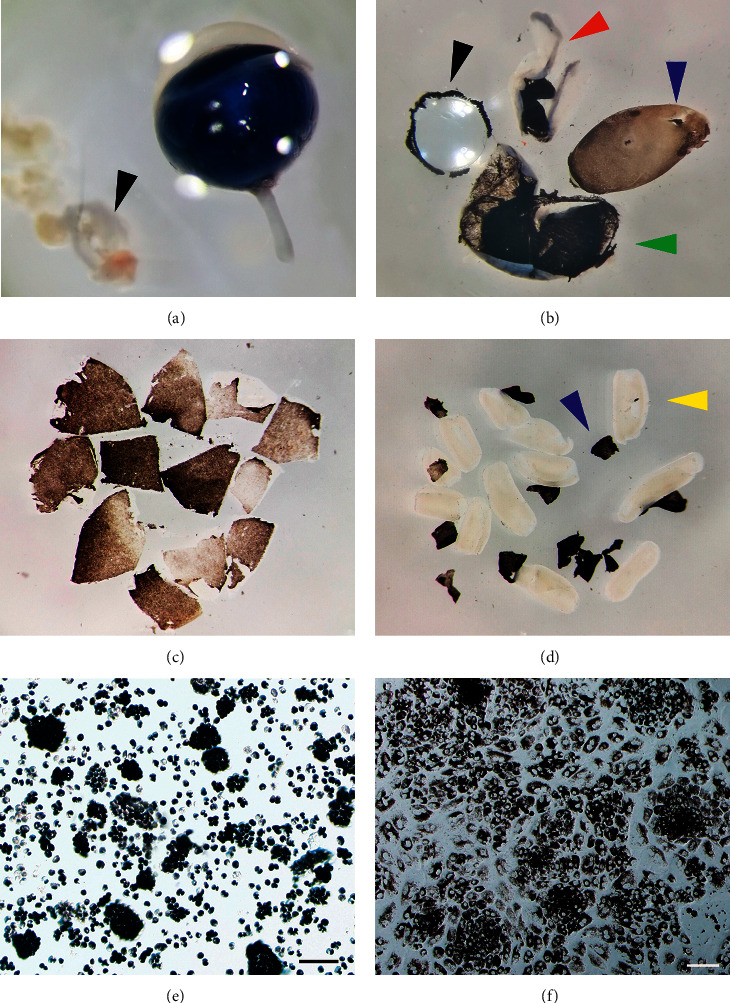
Sequential dissection of DA rat retina to obtain primary RPE cells using dispase. (a) Eyes were dissected out from DA rats, and extraocular muscle attachments (black arrow) were removed from the eye under the dissecting microscope. (b) After incubation in the 2% dispase solution, eyes were washed in PBS. Then the retina/RPE complex (blue arrow) was isolated by removing the choroid (green arrow), the anterior cornea (red arrow), and lens (black arrow). The RPE monolayer was still attached to the retina. (c, d) The resulting retina/RPE complex was cut to small pieces and was further incubated in 10% FBS containing medium for 20 min at 37°C to allow the RPE (blue arrow) to detach from the neural retina (yellow arrow). (e) The detached RPE sheets were collected and digested in 0.1% trypsin solution and then gently pipetted up and down several times to achieve smaller patches of RPE cells. (f) Primary RPE cells of DA rats after 24 h on cell culture dish show high confluence and pigmentation. Scale bar: 100 *μ*m.

**Figure 2:**
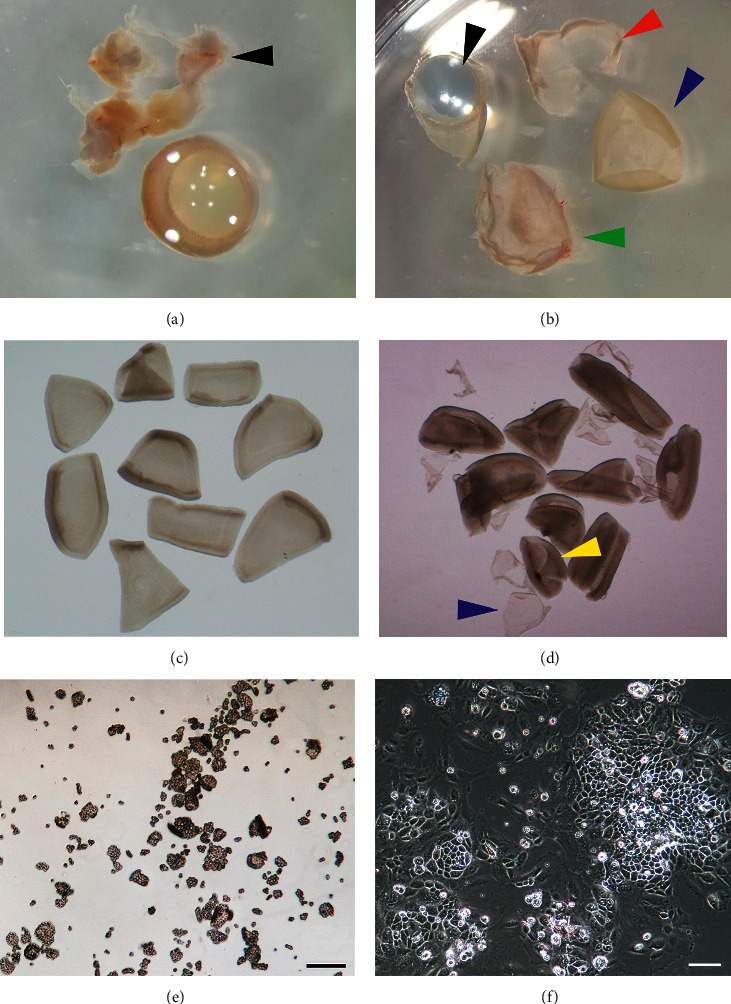
Sequential steps of SD rat eye dissection to obtain primary RPE cells using dispase. (a) Eyes were dissected out from SD rats, and excess muscle attachments (black arrow) were removed in PBS. (b) After incubation in the 2% dispase solution, eyes were washed in PBS. Under dissecting microscope, the retina/RPE complex (blue arrow) was carefully isolated by removing the choroid (green arrow), cornea (red arrow), and lens (black arrow). (c, d) The resulting retina/RPE complex was cut to small pieces and was further incubated in 10% FBS containing medium for 20 min at 37°C to allow the RPE (blue arrow) to detach from the neural retina (yellow arrow). (e) The detached RPE sheets were collected and digested in 0.1% trypsin solution and then gently pipetted up and down several times to achieve smaller patches of RPE cells. (f) Primary RPE cells of SD rats after 24 h on cell culture dish show high confluence and pigmentation. Scale bar: 100 *μ*m.

Rats were killed by CO_2_ asphyxiation. Eyes were enucleated, and extraocular tissues were removed in PBS by using 10 cm suturing forceps (53671D, VISION TECH Co., China) and 8-cm vannas scissors (54140B, VISION TECH Co., China) (black arrows in Figures 1(a) and 2(a)). Then the eyes were incubated in a solution of 2% dispase in DMEM for 30 min at 37°C in a 35 mm dish. Afterwards, dispase solution was removed and eyes were transferred to the growth medium (DMEM/F12 containing 10% FBS) in a 35 mm cell culture dish (Costar, Corning, NY, USA). Under dissecting microscope, a hole was made in the globe just posterior to the ora serrata by using a needle (21 gauge, BD Microlance). Then an incision was made along the ora serrata, and the cornea, lens, and vitreous were discarded (black and red arrows in Figures 1(b) and 2(b)). With the aid of a dissecting microscope, the neural retina with the adherent RPE (retina/RPE complex, blue arrows in Figures 1(b) and 2(b)) was carefully lifted from the slightly attached choroid and sclera (green arrows in Figures 1(b) and 2(b)). Then the retina/RPE complex was cut to small pieces and was further incubated in growth medium for 20 min at 37°C to allow the RPE sheets to detach from the neural retina. During the incubation period, the dish was swirled in a clockwise or counterclockwise direction, which could facilitate the separation of RPE sheets from the retina. At the end of this period, most RPE sheets had detached from the retina spontaneously (blue arrows in Figures 1(d) and 2(d) are pointing towards RPE cells, while yellow arrows are pointing towards the retina), and there is no need to peel RPE sheet from the retina with fine forceps manually.

The detached RPE sheets were collected and washed three times using PBS. Care was taken to avoid collecting retina debris and choroid in each transferring step after each washing. The isolated RPE sheets were collected and digested in 0.1% trypsin solution for 1 min at 37°C. The cells were then suspended in fresh growth medium and gently pipetted up and down several times to achieve smaller patches of RPE cells. The cell solution was diluted with growth medium and washed through centrifugation. RPE cells were resuspended with growth medium and plated (5,000 cells/cm^2^ and 1,5000 cells/cm^2^) on 35 mm cell culture dishes. Medium was changed every two days.

To analyze the effect of culture conditions on RPE morphology and the expression of RPE cell type-specific genes, freshly isolated RPE cells were seeded in either cell culture dishes (35 mm) or petri dishes (35 mm, Baidefu, Haimen, China), at a density of 1,5000 cells/cm^2^ with 10% FBS containing medium. The next day, both the cell culture dish group and the petri dish group were further subgrouped. In half of the dishes in each group, the old media were replaced with fresh 10% FBS containing medium and the other half with N2 and B27 containing medium. Therefore, primary RPE cells were divided into four groups: (1) in cell culture dishes with 10% FBS containing medium (group1, CC dish-FBS), (2) in cell culture dishes with N2 and B27 containing medium (group2, CC dish-N2B27), (3) in petri dishes with 10% FBS containing medium (group3, Petri dish-FBS), and (4) in petri dishes with N2 and B27 containing medium (group4, Petri dish-N2B27). Medium was changed every two days.

### 2.4. Cell Proliferation Assay

Primary RPE cells were seeded in either cell culture dishes (35 mm) or petri dishes (35 mm), at a density of 1,5000 cells/cm^2^ with 10% FBS containing medium. At days 1, 3, 7, 10, and 14, cells in each group were washed twice with PBS and dissociated into single cells with 0.25% trypsin. Cell numbers in each group were measured with the Automated Cell Counter (Shanghai Ruiyu Biotech Co. China).

### 2.5. Quantitative Real-Time PCR (QPCR)

The total RNAs of freshly harvested RPE cells or *in vitro* cultured RPE cells were extracted with Trizol reagent (Takara, Dalian, China), and the cDNAs were reverse transcribed with PrimeScriptTM RT Master Mix (Takara, Dalian, China). QPCR was performed using SuperReal qPCR PreMix (Tiangen Biotech, Beijing, China), and assays were run on a sequence-detection system (Bio-Rad, Hercules, CA, USA). All primer sequences are listed in Table 1. mRNA amplification was normalized against GAPDH.

### 2.6. Western Blot

Freshly harvested RPE cells or *in vitro* cultured RPE cells were collected and lysed in RIPA buffer (P0013, Beyotime, Jiangsu, China) supplemented with protease Inhibitor cocktail (Millipore, 539134-1SML). Protein concentrations were determined by protein assay kit (Thermo Scientific, Rockford, IL, USA), and equal amounts of protein (20 *μ*g each sample) were loaded to 10% SDS-PAGE gels and transferred electrophoretically onto a PVDF membrane (Millipore, Bedford, MA). The membranes were blocked with 5% nonfat milk (Bright Dairy and Food Co., Ltd., Shanghai, China) or 5% BSA (Sangon Biotech, Shanghai, China) at room temperature for 30 minutes. The membranes were then incubated with the primary antibodies against phospho-mTOR (1 : 1,000), total-mTOR (1 : 1,000), phospho-AKT (1 : 1,000), total-AKT (1 : 1,000), phospho-p70S6K (1 : 1,000), total-p70S6K (1 : 1,000), and GAPDH (1 : 5,000), respectively, overnight at 4°C and then followed by incubation with the corresponding secondary antibodies for 1 h at room temperature. After extensive wash, the blots were visualized with a chemiluminescence imaging system (Tanon 5200, Shanghai, China).

### 2.7. Statistical Analysis

The statistical analysis was done with GraphPad Prism software using one-way ANOVA. Data were expressed as mean ± standard deviation (SD), and a *p* value of 0.05 or less was considered statistically significant.

## 3. Results

### 3.1. RPE Cells with Low Density Undergo EMT Losing the Characteristic Epithelial Morphology

**Figure 3:**
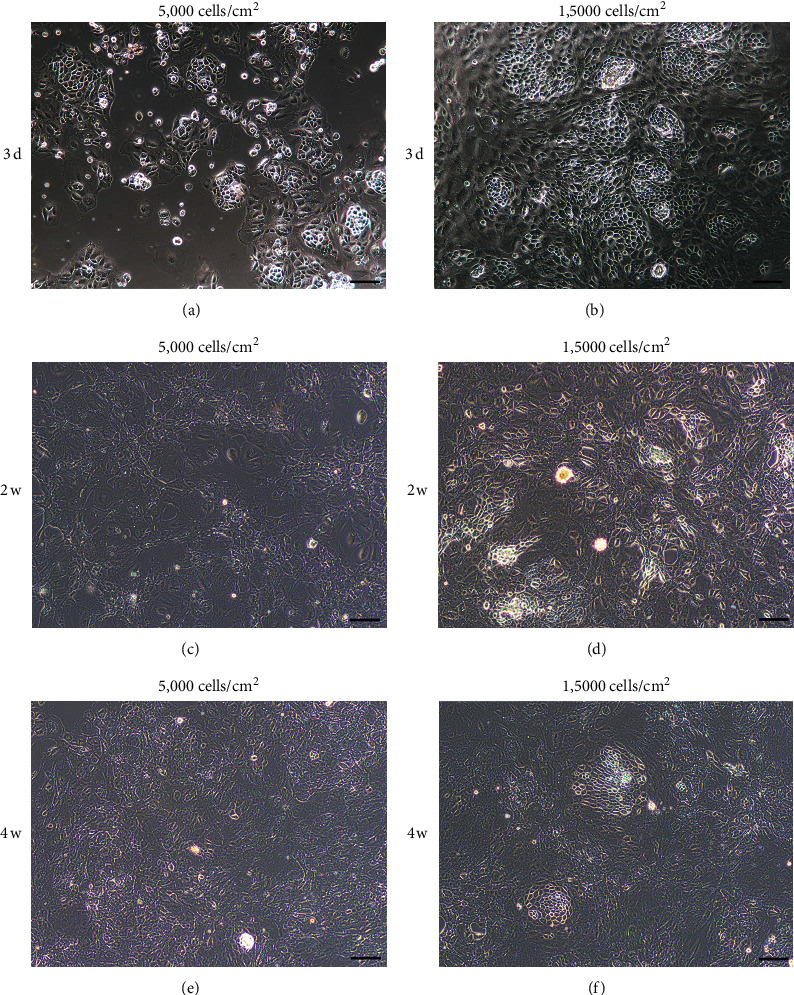
Morphology of the primary RPE cells cultured on cell culture dishes. (a–f) Images of SD rat RPE cells (cell density: 5,000 cells/cm^2^ or 1,5000 cells/cm^2^) cultured on cell culture dishes with 10% FBS containing medium at different time points. Scale bar: 100 *μ*m.

It has been reported that cell density has a profound influence on RPE epithelial phenotype [18]. Therefore, we investigated the effect of initial cell seeding density on the morphology and tissue-specific genes' expression of RPE cells. RPE sheets from SD rats were isolated and plated at either low (5,000 cells/cm^2^) or high (15,000 cells/cm^2^) density. Three days later, RPE cells of both groups demonstrated the typical cobble-stone morphology in the center of the RPE sheets, whereas cells in periphery became flattened and started to migrate away from the sheets (Figures 3(a) and 3(b)). By 2 weeks of culture, RPE cells in the low density group proliferated and migrated to form an almost complete monolayer (Figure 3(c)). However, in the high density group, most RPE cells maintained the differentiation status and packed tightly as a monolayer in culture (Figure 3(d)). At 1 month, the migrating cells in the low density group transitioned to a flattened, elongated fibroblast-like morphology (Figure 3(e)). This phenomenon was also observed in the high density group but with less EMT (Figure 3(f)). These data suggest that plating RPE cells at high density can maintain their hexagonal morphology for at least 1 month in culture.

**Figure 4:**
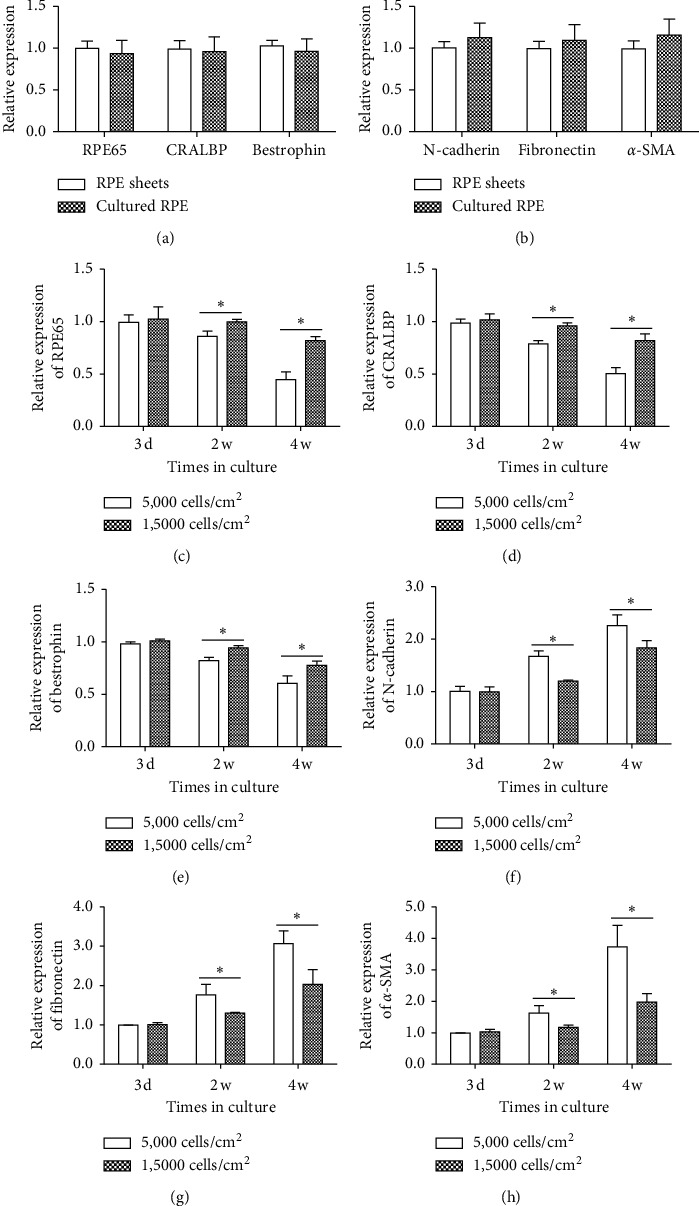
Expression levels of RPE cell type-specific genes and EMT-related genes in SD rat RPE cells at different time points. (a, b) QPCR analysis of RPE65, CRALBP, bestrophin, N-cadherin, fibronectin, and *α*-SMA in freshly isolated RPE tissues and in RPE cells cultured in 10% FBS containing medium (cell density: 1,5000 cells/cm^2^) for 3 days. (c–h) The expressions of RPE65, CRALBP, bestrophin, N-cadherin, fibronectin, and *α*-SMA genes in the two cell density groups at different time points. Low cell density group: 5,000 cells/cm^2^. High cell density group: 1,5000 cells/cm^2^. Data were expressed as mean ± SD. *n* = 4 in each group. ^*∗*^*p* < 0.05.

The loss of polygonal morphology in RPE cells suggested that these cells may lose their differentiated phenotype and undergo EMT. To confirm this, the levels of RPE-specific genes and EMT-related genes were detected. First, we analyzed the expression of RPE-selective genes and EMT-related genes in freshly isolated RPE tissues and *in vitro* cultured RPE cells. After being cultured *in vitro* in a short period (3 days), the levels of RPE65, CRALBP, and bestrophin did not change between the two groups, while the expressions of N-cadherin, fibronectin, and *α*-SMA increased, though not to a significant level (Figures 4(a) and 4(b)). We further analyzed the expression of these genes in the low density group and in the high density group at various time points. As shown in Figures 4(c)–4(e), the expressions of RPE65, CRALBP, and bestrophin decreased markedly at 2 and 4 weeks compared with that at 3 days in culture in both groups. Compared with low density group, these markers were significantly higher in the high density group at 2 and 4 weeks in culture. In contrast, the expressions of EMT markers, including N-cadherin, fibronectin, and *α*-SMA, were increased over time in both groups, with more significance in low density group (Figures 4(f)–4(h)), indicating the EMT happens much easier in low density group.

### 3.2. The Petri Dish-N2B27 Culture System Inhibits EMT of RPE Cells

**Figure 5:**
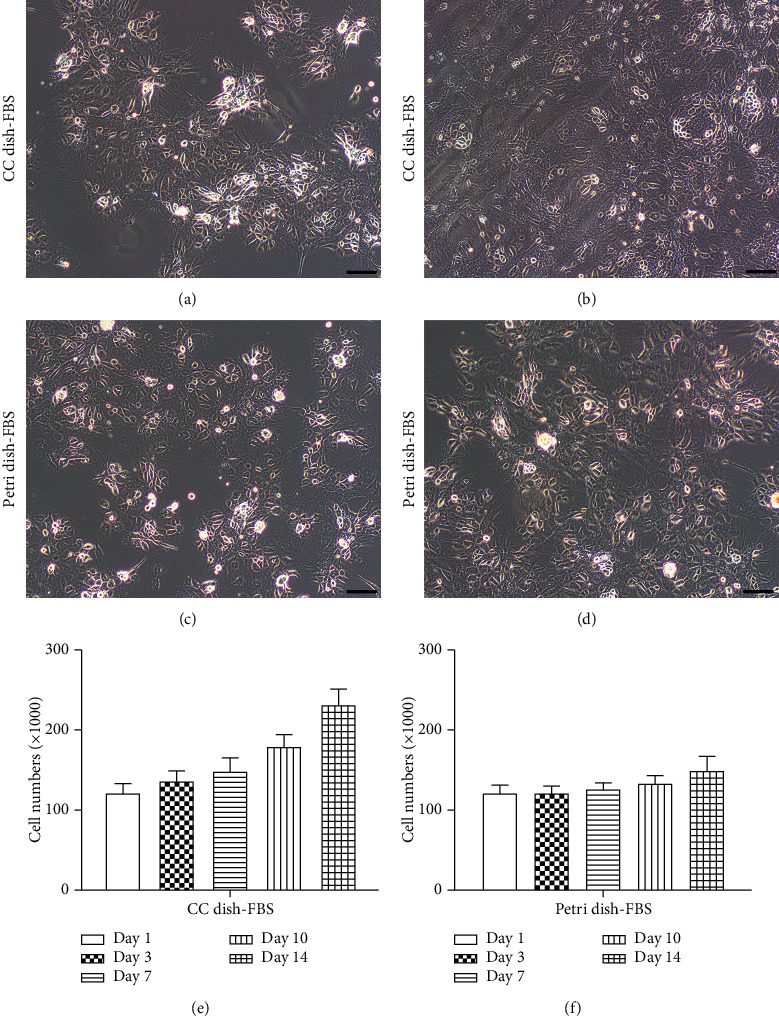
The petri dish culture condition inhibits RPE cell migration and proliferation. (a–d) Images of RPE cells in the cell culture dish group and the petri dish group at different time points. Scale bar: 100 *μ*m. (e) Calculation of RPE cell numbers in the cell culture dish group and the petri dish group at days 1, 3, 7, 10, and 14. Data were expressed as mean ± SD. *n* = 4 in each group.

**Figure 6:**
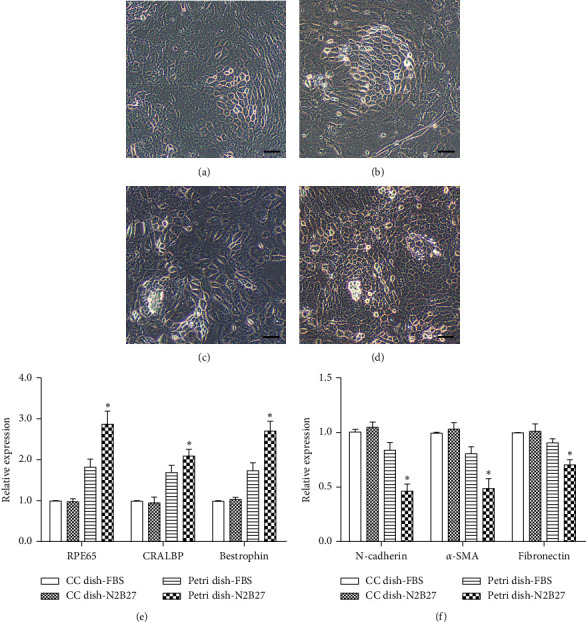
Characteristics of SD rat RPE cells under different *in vitro* culture conditions. (a–d) Images of RPE cells cultured in different culture systems at 1 month. Scale bar: 100 *μ*m. (e) QPCR analysis of RPE65, CRALBP, and bestrophin expressions in RPE cells from SD rats at 1 month in different culture conditions. (f) Expression levels of N-cadherin, fibronectin, and *α*-SMA in SD rat RPE cells at 1 month in different culture conditions. Cell density: 1,5000 cells/cm^2^. Data were expressed as mean ± SD. *n* = 4 in each group. ^*∗*^*p* < 0.05, the petri dish-N2B27 group versus the other three groups at 1 month in four culture conditions. (a) CC dish-FBS. (b) CC dish-N2B27. (c) Petri dish-FBS. (d) Petri dish-N2B27.

It becomes a routine to cultivate primary cells and cell lines in FBS containing medium in cell culture dishes [16]. The undefined components of FBS and the surface properties of cell culture dishes might affect cell proliferation, adhesion, and migration, thus contributing a pivotal role for the subsequent initiation of EMT in RPE cells. To explore an ideal culture system and avoid EMT for the primary RPE cells, we cultivated primary RPE cells in petri dishes and replaced serum with N2 and B27 supplements in the culture system. By 2 weeks of culture, RPE cells in the cell culture dish group proliferated and migrated to form a monolayer, while RPE cells in the petri dish group did not proliferate (Figures 5(a)–5(d)). RPE cells grown in the cell culture dish had increasing linear growth with time while cell proliferation was strictly inhibited in the petri dish group (Figure 5(e)). As shown in Figure 6, RPE cells in the cell culture dish condition, supplemented with either FBS or N2B27, transitioned to a fusiform morphology with few hexagonal cells visible in central regions of the original RPE sheets (Figures 6(a) and 6(b)). In the petri dish-FBS group, the cell proliferation was inhibited, but the RPE cells failed to retain an epithelial morphology and became flattened and elongated (Figure 6(c)). In contrast, the RPE cells maintained an epithelial morphology and tightly packed as a monolayer in the petri dish-N2B27 group (Figure 6(d)), indicating the petri dish-N2B27 condition might be the optimal condition for the primary RPE cells cultivation. To validate this, both RPE cell type-specific genes and EMT-related genes were examined with Q-PCR. And the data showed that the petri dish-N2B27 culture condition upregulated the expressions of RPE65, CRALBP, and bestrophin and downregulated EMT-related markers including N-cadherin, fibronectin, and *α*-SMA (Figures 6(e) and 6(f)). From the data above, the petri dish-N2B27 condition is more suitable for the culture of primary RPE cells and maintains their epithelial state.

**Figure 7:**
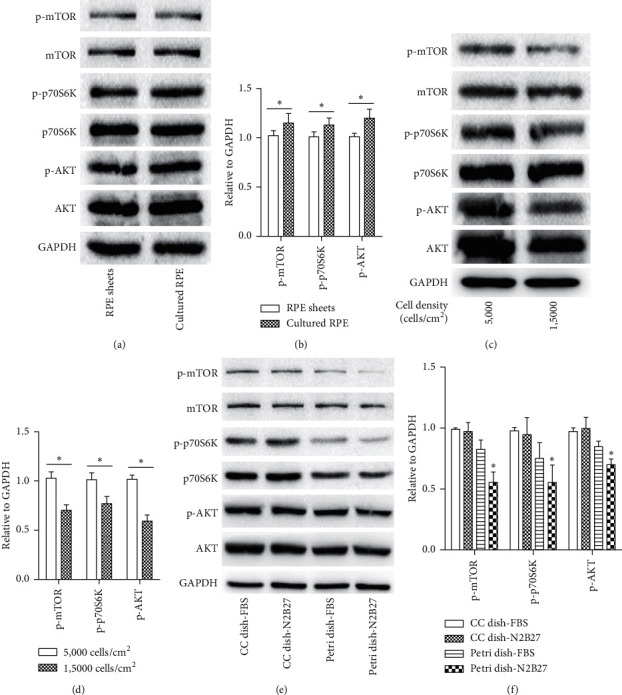
The petri dish-N2B27 culture condition inhibits AKT/mTOR pathway activation. (a) Protein levels of phospho-mTOR, total-mTOR, phospho-p70S6K, total-p70S6K, phospho-AKT, and total-AKT in freshly isolated RPE tissues and in RPE cells cultured in 10% FBS containing medium (cell density: 1,5000 cells/cm^2^) for 3 days. (b) Quantification of the western blot data from (a). (c) The expressions of phospho-mTOR, total-mTOR, phospho-p70S6K, total-p70S6K, phospho-AKT, and total-AKT in the two cell density groups at different time points. Low cell density group: 5,000 cells/cm^2^. High cell density group: 1,5000 cells/cm^2^. (d) Quantification of the western blot data from (c). Data were expressed as mean ± SD. *n* = 4 in each group. ^*∗*^*p* < 0.05. (e) Western blot analysis of phospho-mTOR, total-mTOR, phospho-p70S6K, total-p70S6K, phospho-AKT, and total-AKT in SD rat RPE cells cultured in different culture conditions. Cell density: 1,5000 cells/cm^2^. (f) Quantification of the western blot data from (e). Data were expressed as mean ± SD. *n* = 4 in each group. ^*∗*^*p* < 0.05, the petri dish-N2B27 group versus the other three groups at 1 month in four culture conditions.

mTOR pathway activation is vital for the regulation of RPE cell migration and proliferation. Therefore, we characterized the mTOR pathway in rat RPE cells cultured in the four culture systems. After 4 weeks in the CC dish-FBS condition, the protein levels of the major components of the mTOR signaling networks including phospho-mTOR, phospho-p70S6K, and phospho-AKT were evidently higher in *in vitro* cultured RPE group than those in the freshly isolated RPE group (Figures 7(a) and 7(b)). As for the effect of the initial cell seeding density on the activation of mTOR signaling, the expressions of the mTOR signaling proteins in the low density group were markedly higher than that in the high density group (Figures 7(c) and 7(d)). When RPE cells were cultured in the petri dish-N2B27, the protein levels of phospho-mTOR, phospho-p70S6K, and phospho-AKT were decreased significantly compared with those in the other three groups (Figures 7(e) and 7(f)).

## 4. Discussion

RPE cells exert many essential functions in the visual cycle, and their dysfunction would lead to retinal degeneration and vision impairment. The RPE cell has an apical to basolateral polarity by structure, characterized by the presence of apical microvilli and basal infoldings. This, as well as its barrier function that inhibits drug molecules from passing from the blood to the retina, makes experimentation with RPE difficult *in vivo*. Thus, the primary RPE cell culture serves as a valuable *in vitro* model to study RPE transport, protein localization and function, and the side-effects of drugs on RPE cells. However, when RPE cells are isolated and cultured *in vitro*, they are exposed to various cytokines and growth factors, and the interactions of cell-cell and cell-matrix are largely altered under such conditions, which contribute to the subsequent initiation of EMT in RPE cells. Therefore, it is of great importance for successful isolation of the primary RPE cells with more efficiency as well as maintaining its epithelial characteristics for *in vitro* characterization.

The previous methods for RPE cell isolation include mechanical dissection techniques and enzymatic dissociation [14, 19, 20]. Although these methods are useful for RPE cell isolation and culture, difficulties still arise as to maintain the hexagonal, epithelioid monolayer that better mimics native RPE cells. In this study, we provide an alternative method for isolation and culture of RPE cells which includes first enzyme incubation and then followed the isolation and culture of RPE cells. Dispase incubation allows the RPE monolayer to remain attached to the neural retina during isolation of the retina (Figures 1(b) and 2(b)), which greatly reduces the risk of contamination of the cells from the choroids. In the previous methods for RPE isolation [14, 21], the retina/RPE complex is incubated in the enzyme solution or the growth medium as a whole, and the RPE sheets need to be peeled off the retina manually, which is labor-intensive and time-consuming. More importantly, this approach is ineffective for the isolation of albino rat RPE cells because they are not pigmented. In contrast, our method is much easier and time-saving because the RPE sheets could detach from the neural retina spontaneously during the incubation period (Figures 1(d) and 2(d)). Once cut into small pieces, the neural retina would curl inwardly incubated in the growth medium, which facilitated the detachment of RPE sheet in the medium (Figures 1(d) and 2(d)). This is especially important for the isolation of RPE cells from albino rats since the RPE monolayer is transparent, which prevents the direct observation of the nonpigmented RPE, and the separation of RPE sheets from the neural retina with fine forceps can be ambiguous. This easier manipulation provides a rapid and efficient method for isolating RPE cells for further studies.

Primary RPE cell culture has been practiced for decades, and one problem that confuses many researchers is that *in vitro* cultured RPE cells easily undergo EMT. It has been demonstrated that the contacts between cells are important in the maintenance of RPE phenotype, and the disruption of such contacts has been associated with the initiation of EMT [22]. When RPE cells are isolated and cultured *in vitro*, the disruption of the cell-cell and cell-matrix interactions promotes their proliferation and EMT [18, 23]. Many factors have been associated with the process of EMT, such as cell seeding density and culture conditions. For example, plating RPE cells in low density resulted in RPE dedifferentiation, whereas plating RPE cells at higher density maintained their morphology and characteristic gene expressions for a long period [14]. This is in agreement with our observation that RPE cell morphology and RPE cell characteristic gene expressions were influenced by the initial cell density. When plated with low density (5,000 cells/cm^2^), the cultured RPE cells underwent EMT with enlarged and irregular cell morphology and increasing EMT-related markers, while losing RPE-specific markers, such as CRALBP and RPE65, over time. The above was largely avoided when plating RPE cells with high density, that is, 15,000 cells/cm^2^ (Figures 3 and 4).

The *in vitro* cultured RPE cells also experience changes with regard to exposure to different culture medium, growth factors and culture dishes. For instance, FBS contains a wide variety of hormones, cytokines, and growth factors, and DMEM containing FBS might be not maintain the epithelial state of RPE cells during the culture [24]. To avoid EMT, we choose N2 and B27 supplements to support the maintenance and growth of RPE cells. N2 and B27 are commercially formulated and contain a number of factors that have been shown to affect RPE phenotype [16, 17]. In recent studies on the possible mechanism of mesenchymal-epithelial transition (MET), petri dish-N2B27 condition was demonstrated as an effective method to reverse dedifferentiated phenotype of RPE cells into epithelialized phenotype [25]. In our work, we found that the petri dish condition is also very important for the maintenance of the epithelialized phenotype of rat RPE cells. Petri dishes are untreated and have a hydrophobic surface, which inhibits cell adhesion and spreading. As a result, the migration and proliferation of RPE cells are strictly inhibited in the petri dish condition, which helps maintain its phenotype and inhibits EMT. When RPE cells were cultured in petri dishes rather than cell culture dishes, EMT was largely prevented, even in FBS containing medium. Since the petri dish inhibits cell spreading, the proliferation and migration of RPE cells were strictly inhibited (Figure 5). Most of the cells in the petri dish-N2B27 group maintained cobblestone-like morphology with upregulated RPE-selective genes and downregulated EMT-related genes (Figure 6). These findings suggested that the petri dish containing N2B27 could well maintain the epithelial condition of RPE and inhibit its EMT *in vitro*.

Interestingly, we also found that RPE cell proliferation and EMT coincided with the robust activation of AKT/mTOR pathway. The AKT/mTOR pathway modulates EMT process in multiple ways [26, 27]. For instance, AKT/mTOR signaling regulates the detrimental dedifferentiation and hypertrophy of RPE cells through reduction of RPE cell type-specific proteins [28]. In the present study, we also examined AKT/mTOR pathway in RPE cells in four different culture conditions and found that it was evidently inhibited in the RPE cells when cultured in the petri dish condition (Figure 7). However, we found that when RPE cells were cultured in the petri dish-FBS condition, even though mTOR signaling was inhibited, EMT was still activated with downregulated RPE-selective genes and upregulated EMT-related genes (Figures 6 and 7). This finding suggests that the petri dish condition is necessary but not sufficient for EMT inhibition. N2 and B27 are also necessary for EMT inhibition of RPE cells *in vitro*.

In summary, we presented a novel and reliable method for the primary RPE cell isolation using dispase. Compared with other already published protocols, this method is easy to manipulate and time-saving, which avoids the contamination from the choroids. In addition, our data suggested that the petri dish-N2B27 culture system is much more suitable for primary RPE cells cultivation, which might be well mimic the *in situ* condition for further study.

## Figures and Tables

**Table 1:** Primer information for QPCR.

Genes	Forward sequence (5′-3′)	Reverse sequence (5′-3′)	Accession no.
RPE65	ACCTCTTCCATCACATCAATAC	CTTCCCAGTTCTCACGTAAAT	XM_003127931.4
CRALBP	CTCCCGTGAGCCTGATTGAA	CCTTGACCGTCCCTGATAGC	NM_001106274.1
Bestrophin	TCTTCCTGCTCTGCTACTAC	GGATGTAGCTGTCGCAATAC	XM_005660805.2
N-cadherin	AAGGTGGATGAGGACGGCAC	ACAGCTACCTGCCACTTTTCC	NM_031333.1
Fibronectin	GAGACCACCATCACCATTAG	GTTCTCTGGATTGGAGTCTG	AY839862.1
*α*-SMA	GATCTGGCACCACTCTTTC	CATAATCTGGGTCATCTTCTCC	XM_013983522.1
GAPDH	ACATTTTGCTGATGACTGGTTACA	GGTGGTGAACTTGTTTTGCGA	AF106860.2

## Data Availability

The data used to support the findings of this study are available from the corresponding author upon request.
